# Anatomical variations in the Circle of Willis and the formation and rupture of intracranial aneurysms: A systematic review and meta-analysis

**DOI:** 10.3389/fneur.2022.1098950

**Published:** 2023-01-16

**Authors:** Lu Feng, He-Jiao Mao, Ding-Ding Zhang, Yi-Cheng Zhu, Fei Han

**Affiliations:** ^1^Department of Neurology, Peking Union Medical College Hospital, Chinese Academy of Medical Sciences and Peking Union Medical College, Beijing, China; ^2^Central Research Laboratory, Peking Union Medical College Hospital, Chinese Academy of Medical Sciences and Peking Union Medical College, Beijing, China

**Keywords:** Circle of Willis, intracranial aneurysm, variation, rupture, anterior communicating artery, posterior communicating artery

## Abstract

**Background:**

The anterior (AcomA) and posterior communicating arteries (PcomA) are two of the most frequent sites for intracranial aneurysms. Anatomical variations in the Circle of Willis (COW) are frequently observed in patients with AcomA and PcomA aneurysms. Strong evidence is needed to determine the pooled estimate of the effect of COW variations on the formation and rupture of these aneurysms.

**Aim:**

This systematic review and meta-analysis aimed to establish the effect of COW variations on the formation and rupture of AcomA and PcomA aneurysms using available studies.

**Summary of review:**

PubMed, Embase, and Web of Science databases were systematically searched for studies published in English before September 21, 2022. Studies investigating AcomA aneurysms and the hypoplastic/aplastic A1 segment of the anterior cerebral artery and PcomA aneurysms and hypoplastic/aplastic PcomA or fetal-type posterior cerebral artery (FTP) were included. The heterogeneity of the studies was assessed using Cochran *Q*-test and I^2^ statistic. Pooled estimate was assessed using either a random- or fixed-effects model based on the heterogeneity of the studies. Among the 4,932 studies, 21 were eligible and included in the analysis. The presence of hypoplastic/aplastic A1 was significantly correlated with the formation [OR (95% confidence interval [CI]) = 7.97 (5.58, 11.39), *P* < 0.001] and rupture [OR (95%CI) = 1.87 (1.29, 2.72), *P* < 0.001] of AcomA aneurysms. Significant associations between FTP and both the formation [OR (95%CI) = 2.15 (1.41, 3.30), *P* < 0.001] and rupture [OR (95%CI) = 1.72 (1.26, 2.36), *P* < 0.001] of PcomA aneurysms were observed.

**Conclusions:**

Significant associations were observed between COW variations and both the formation and rupture of AcomA and PcomA aneurysms. This can help in determining interventions for patients with aneurysms.

**Systematic review registration:**

https://www.crd.york.ac.uk/prospero/display_record.php?RecordID=225149, identifier: CRD42021225149.

## Introduction

Intracranial aneurysms are acquired abnormal vascular dilations that occur in 1–2% of the population (1). The most dangerous complications of intracranial aneurysms are their rupture, which accounts for 80–85% of non-traumatic subarachnoid hemorrhages with high mortality and disability rates (1). Various risk factors have been identified for the rupture of

intracranial aneurysms, including age, aneurysm size, and location (2–4). The anterior (AcomA) and posterior communicating arteries (PcomA) are two of the most frequent sites for intracranial aneurysms (3), where ruptures are more likely to occur than at other sites (5).

Hemodynamics plays an important role in the growth and rupture of intracranial aneurysms (6). The Circle of Willis (COW) is a vascular network located at the base of the brain with a high variation rate (7). COW variations can cause hemodynamic changes by influencing cerebral blood flow; moreover, they are proposed to be contributors to the development of intracranial aneurysms (8, 9).

Hypoplastic/aplastic A1 and fetal-type posterior cerebral artery (FTP) are commonly observed in patients with AcomA and PcomA aneurysms, respectively. A series of studies have assessed the association between hypoplastic/aplastic A1 and the formation of AcomA aneurysms (10–13); meanwhile, the evidence on FTP and the formation of PcomA aneurysms is insufficient (14–16). In addition, there is a lack of consistency in the association between COW variations and rupture of AcomA and PcomA aneurysms (17–20).

Strong evidence is needed to determine the pooled estimate of the effect of COW variations on the formation and rupture of site-specific aneurysms. This will serve as evidence of the effect of hemodynamic factors in the development of intracranial aneurysms and may facilitate the prediction of rupture risk of observed aneurysms. Thus, this systematic review and meta-analysis aimed to establish the effect of COW variations on the formation and rupture of AcomA and PcomA aneurysms using available studies.

## Methods

This systematic review and meta-analysis was conducted in accordance with the Preferred Reporting Items for Systematic Reviews and Meta-Analyses (PRISMA). The protocol for this meta-analysis was registered at https://www.crd.york.ac.uk/prospero/display_record.php?RecordID=225149.

### Search strategy

Relevant studies published before September 21, 2022 were selected by searching PubMed, Embase, and Web of Science databases. A search strategy was developed that combines both free text words and medical subject headings covering, “intracranial aneurysm,” “variation,” and “Circle of Willis.” The detailed search strategy was listed in Supplementary Tables 1–3. The citations of the included reviews and articles were manually searched to obtain additional relevant studies.

### Study selection

Studies that satisfied the following criteria were included: (1) investigated the relationship of COW configuration and formation or rupture of AcomA and PcomA aneurysms; (2) were cross-sectional, case-control, or cohort studies; (3) reported odds ratios (ORs) with 95% confidence intervals (CIs) (or reported data that was sufficient to calculate ORs and 95% CIs); and (4) published in English.

The exclusion criteria were as follows: (1) were case reports, editorials, conference abstract, or letters; and (2) were model/computer-generated brain studies. If the study population overlapped in two or more papers by the same authors, only the study with the largest number of participants were included.

### Data extraction and study quality evaluation

Two investigators (LF and H-JM) independently reviewed the study titles and abstracts, and studies that satisfied the inclusion criteria underwent full-text assessment. Data were extracted independently by the two authors according to the pre-designed forms. The extracted data of the selected study included: first author, year of publication, country or area, study design, total number of participants, age, sex, COW variation types and definitions, aneurysm size, imaging techniques, and outcome variables. A consensus was reached through discussion when there were any disagreements.

The Newcastle–Ottawa scale (NOS) was used to assess the quality of cohort and case-control studies (21). The version of the scale consists of three categories: selection, comparability, and outcome. A study can be given a score ranging from zero to nine stars (low-quality: 0–3, medium-quality: 4–6, high-quality: 7–9). The Agency for Healthcare Research and Quality (AHRQ) scale was used to assess the quality of cross-sectional studies (22). A study can be given a score ranging from zero to 11 points (low-quality: 0–3, medium-quality: 4–7, high-quality: 8–11).

### Statistical analysis

The effect measures of interest were ORs and 95% CIs. The heterogeneity of the studies was assessed using the Cochran *Q*-test and I^2^ statistic (23). For the pooling of study results, a random-effects model was used when between-study heterogeneity was statistically significant (*P* < 0.05 or I^2^ > 50%), while a fixed-effects model was applied when the heterogeneity was not significant (*P* > 0.05 and I^2^ <50%). Subgroup analysis was performed based on the locations and types of study. Sensitivity analysis was performed by removing each study in turn and evaluating whether the pooled estimate was affected significantly (24). The Egger test was used to assess publication bias when the included studies were more than 10, and significant publication bias was defined as *p* < 0.1 (25). Statistical analysis was performed using Stata 12.0.

## Results

### Identified studies

**Figure 1:**
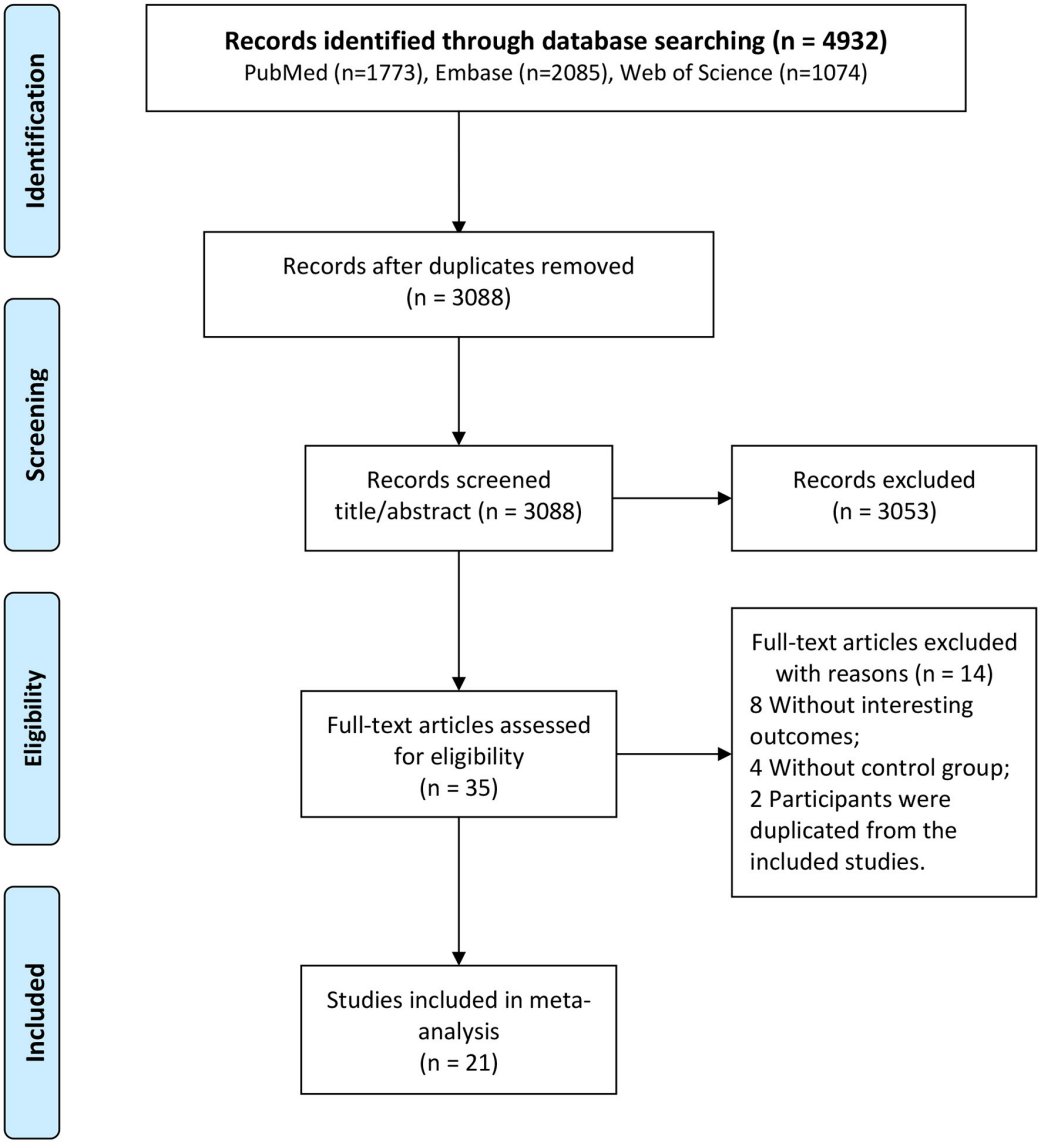
Study selection process.

A total of 4,932 studies were identified after a literature search (1,773 from PubMed, 2,085 from Embase, and 1,074 from Web of Science) (Figure 1). Among these, 1,844 were replicated, 3,053 were excluded after reviewing the title and abstract, and 35 were selected for full-text assessment. Moreover, among these, 14 were excluded, and 21 studies fulfilled the eligibility criteria and were included in the analysis (10–20, 26–35).

### Study characteristics and quality

The 21 studies included in this systematic review and meta-analysis were published between 1980 and 2021 and conducted in China, Japan, Korea, Poland, United States of America, and other countries. A total of six cross-sectional studies, three retrospective cohort studies, and 12 case-control studies were included. A summary of the study characteristics is listed in Tables 1, 2.

**Table 1:** Characteristics of the included studies in this meta-analysis.

**Study**	**Area**	**Design**	**Recruit-ment time**	**Imaging technique**	**Type of variation**	**Variants definition**	**Groups**	**n, M/F**	**Age, years**	**Size of aneurysm, mm**
Charbel (31)	USA	CCS	1976–1988	CA	A1	<50% of the contralateral A1	AcomA-A	51, 32/19	49 (17–77)	NR
							Non-IA	50, 25/25	45 (10–75)	
Chen (10)	China	CSS	2009–2012	MRA	A1	<1 mm or the absence of A1	All	8013, 3660/4353	49.85 (16–89)	NR
							AcomA-A	138, NR	NR	NR
							Non-IA	7875, NR	NR	
de Rooij (31)	The Netherlands	CCS	2000–2006	CTA	A1, FTP, PcomA,	A1/PcomA: <1 mm or not visible FTP: PcomA > 110% P1	Ruptured IA	75, 18/57	52 (18–79)	NR
							Unruptured IA	75, 17/58	54 (26–81)	NR
He (16)	China	CSS	2013–2016	CTA	FTP	PComA > P1	All	364, 218/146	61.73 ± 13.33	NR
							PcomA-A	24, NR	NR	NR
							Other IA	26, NR	NR	NR
							Non-IA	314, NR	NR	
Horikoshi (32)	Japan	CCS	1997–2000	MRA, CTA, DSA	A1	Not visualized or only a very thin segment	All aneurysm	131, 43/88	69.3 (44–85)	NR
							AcomA-A	31, NR	69.3 (44–85)	NR
							Other IA	100, NR	69.3 (44–85)	NR
							Non-IA	434, NR	65.4 (40–89)	
Hu (15)	China	CCS	2008–2013	DSA	FTP	Blood supplied by ICA	PcomA-A	76, 19/57	58.3 ± 11.6	NR
							Non-IA	78, 23/55	56.5 ± 12.1	
Huhtakangas (17)	Finland	CSS	2000–2014	CTA	FTP	Absent or hypoplastic P1 with PCA arising directly from the PComA	PcomA-A	391, 92/299	57 ± 13 (20–92)	NR
							Ruptured PcomA-A	256, 53/203	NR	7.9 ± 3.4
							Unruptured PcomA-A	135, 40/95	NR	5.2 ± 3.5
Jabbarli (27)	Germany	RCS	2005–2012	DSA	A1	NR	Ruptured IA	594, 220/374	55.2 (21–94)	NR
							Ruptured AcomA-A	228, NR	NR	NR
							Unruptured AcomA-A	10, NR	NR	NR
Kaspera (11)	Poland	CCS	2007–2013	CTA	A1	Vascular asymmetry coefficient>40%	AcomA-A	77, 35/42	57 (49–64)	NR
							Non-IA	73, 29/44	50 (38–61)	
Kayembe (34)	Japan	CCS	1976–1982	autopsy	A1, PcomA	NR	All aneurysm	44, 21/23	36–81	NR
							Non-IA	148, NR	NR	
Krasny (12)	Germany	CCS	2002–2010	CA	A1	<3/4 of the contralateral A1	AcomA-A	223, 111/112	54 ± 16	7.30 ± 3.26/6.3 ± 3.08
							Non-IA	204, 105/99	52 ± 20	
Krzyzewski (13)	Poland	CCS	NR	CTA	A1	<1mm	AcomA-A	50, 25/25	53.66 ± 14.01	NR
							Non-IA	100, 50/50	53.47 ± 14.48	
Kwak (35)	Japan	CCS	1961–1975	CA	A1	<50% of the contralateral A1	All aneurysm	485, 279/206	18–72	NR
							AcomA-A	213, NR	18–72	NR
							Non-IA	76, 51/25	4–57	
Lv (28)	China	CSS	2014–2015	CA	FTP	PcomA > P2, with atrophic P1	PcomA-A	108, 24/84	60 (42–82)	4.36 (1.23–6.84)
							Ruptured PcomA-A	68, 13/55	58	4.50
							Unruptured PcomA-A	40, 11/29	62	3.92
Matsukawa (29)	Japan	CSS	2003–2012	MRA, CTA, DSA	FTP	PcomA > P2, with atrophic P1	PcomA-A	134, 92/42	66 ± 13	4 (2.5–6.0)
							Ruptured PcomA-A	39, 30/9	61 ± 14	5.9 (4.0–7.5)
							Unruptured PcomA-A	95, 62/33	68 ± 12	3.4 (2.0–5.0)
Park (19)	Korea	RCS	2016–2020	DSA	A1	<50% of the contralateral A1	AcomA-A	209, 105/104	59.80 ± 11.15	5.44 ± 2.46
							Ruptured AcomA-A	102, 51/51	56.62 ± 11.95	6.10 ± 2.54
							Unruptured AcomA-A	107, 54/53	62.84 ± 9.42^*^	4.81 ± 2.22^*^
Rinaldo (26)	USA	RCS	2003–2013	DSA	A1	<50% of the contralateral A1	AcomA-A	204, 100/104	58.6 ± 13.0	6.3 ± 3.4
							Ruptured AcomA-A	148, 79/69	56.9 ± 12.8	6.1 ± 3.6
							Unruptured AcomA-A	56, 21/35^*^	62.7 ± 11.5^*^	6.5 ± 3.0
Silva Neto (14)	Brazil	CCS	2001–2007	CA	A1, FTP	A1: NR FTP: Not visualized P1 segment	All aneurysm	158, 49/109	48.8 ± 19.2	NR
							Non-IA	256, NR	NR	
Tarulli (30)	Canada	CCS	2005–2009	DSA, CTA, MRA	A1	<50% of the contralateral A1	AcomA-A	105, 52/53	52 ± 11	NR
							Other IA	123, 31/92	50 ± 11	NR
							Non-IA	159, 75/84	69 ± 15	
Xu (18)	China	CSS	2009–2016	DSA	FTP	Not visualized P1 segment	Ruptured PcomA-A	89, 9/80	62.1 ± 11.7	6.91 ± 4.12
							Unruptured PcomA-A	166, 28/138	59.2 ± 9.6	5.42 ± 2.57^*^
Zhang (20)	China	CCS	2008–2020	DSA, CTA, MRA	A1	<50% of the contralateral A1	AcomA-A	253, 137/116	54.6 ± 12.7	4.38 ± 1.98
							Ruptured AcomA-A	218, 118/100	NR	4.56 ± 1.96
							Unruptured AcomA-A	35, 19/16	NR	3.24 ± 1.79^*^
							Non-IA	83, NR	52.7 ± 13.1	

AcomA, anterior communicating artery; AcomA-A, anterior communicating artery aneurysm; CA, cerebral angiography; CCS, case-control study; CSS, cross-sectional study; CTA, computed tomography angiography; DSA, digital subtraction angiography; F, female; FTP, fetal-type posterior cerebral artery; IA, intracranial aneurysm; M, male; MRA, magnetic resonance angiography; NR, not reported; PcomA, posterior communicating artery; PcomA-A, posterior communicating artery aneurysm; RCS, retrospective cohort study.

^*^P < 0.05.

**Table 2:** Summary of the case group, control group, and COW variations of the included studies.

**Study**	**Design**	**Variation**	**Groups**	**N**	**Yes**	**No**
Charbel (31)	CCS	A1	AcomA aneurysm	37	9	28
			Non-IA	50	3	47
Chen (10)	CSS	A1	AcomA aneurysm	138	60	78
			Non-IA	7,875	365	7,510
de Rooij (31)	CCS	A1	Ruptured AcomA aneurysm	11	3	8
			Unruptured AcomA aneurysm	11	4	7
		PcomA	Ruptured PcomA aneurysm	10	6	4
			Unruptured PcomA aneurysm	10	7	3
		FTP	Ruptured PcomA aneurysm	10	4	6
			Unruptured PcomA aneurysm	10	3	7
He (16)	CSS	FTP	PcomA aneurysm	24	11	13
			Other IA	26	5	21
			Non-IA	314	75	239
Horikoshi (32)	CCS	A1	AcomA aneurysm	31	19	12
			Other IA	100	14	86
			Non-IA	434	67	367
Hu (15)	CCS	FTP	PcomA aneurysm	152	26	126
			Other IA	156	15	141
Huhtakangas (17)	CSS	FTP	Ruptured PcomA aneurysm	258	70	188
			Unruptured PcomA aneurysm	155	27	128
Jabbarli (27)	RCS	A1	Ruptured AcomA aneurysm	228	106	122
			Unruptured AcomA aneurysm	10	1	9
Kaspera (11)	CCS	A1	AcomA aneurysm	77	33	44
			Non-IA	73	9	64
Kayembe (34)	CCS	A1	AcomA aneurysm	27	9	18
			Other IA	17	1	16
			Non-IA	146	16	130
		PcomA	PcomA aneurysm	8	5	3
			Other IA	36	11	25
			Non-IA	134	22	112
Krasny (12)	CCS	A1	AcomA aneurysm	223	141	82
			Non-IA	204	48	156
Krzyzewski (13)	CCS	A1	AcomA aneurysm	50	18	32
			Non-IA	100	9	91
Kwak (35)	CCS	A1	AcomA aneurysm	213	145	68
			Non-IA	76	22	54
Lv (28)	CSS	FTP	Ruptured PcomA aneurysm	68	20	48
			Unruptured PcomA aneurysm	40	11	29
Matsukawa (29)	CSS	FTP	Ruptured PcomA aneurysm	39	17	22
			Unruptured PcomA aneurysm	95	30	65
Park (19)	RCS	A1	Ruptured AcomA aneurysm	102	58	44
			Unruptured AcomA aneurysm	107	42	65
Rinaldo (26)	RCS	A1	Ruptured AcomA aneurysm	148	28	120
			Unruptured AcomA aneurysm	56	6	50
Silva Neto (14)	CCS	A1	AcomA aneurysm	59	28	31
			Other IA	99	1	98
			Non-IA	256	7	249
		FTP	PcomA aneurysm	59	14	45
			Other IA	99	2	97
			Non-IA	256	33	223
Tarulli (30)	CCS	A1	AcomA aneurysm	105	73	32
			Other IA	123	14	109
			Non-IA	159	37	122
Xu (18)	CSS	FTP	Ruptured PcomA aneurysm	89	27	62
			Unruptured PcomA aneurysm	166	28	138
Zhang (20)	CCS	A1	AcomA aneurysm	253	141	112
			Non-IA	83	10	73
		A1	Ruptured AcomA aneurysm	218	169	84
			Unruptured AcomA aneurysm	35	14	69

AcomA, anterior communicating artery; CCS, case-control study; CSS, cross-sectional study; FTP, fetal-type posterior cerebral artery; IA, intracranial aneurysm; PcomA, posterior communicating artery; RCS, retrospective cohort study.

Overall, the included cross-sectional studies that were assessed using the AHRQ scale received 6–9 scores, while the cohort and case-control studies that were assessed using NOS received 6–7 and 5–7 scores, respectively. Five studies were of high quality (15, 19, 20, 26, 29), while the others were of medium quality. A summary of the study quality assessment is provided in Supplementary Tables 4–6.

### Hypoplastic/aplastic A1 and AcomA aneurysms

**Figure 2:**
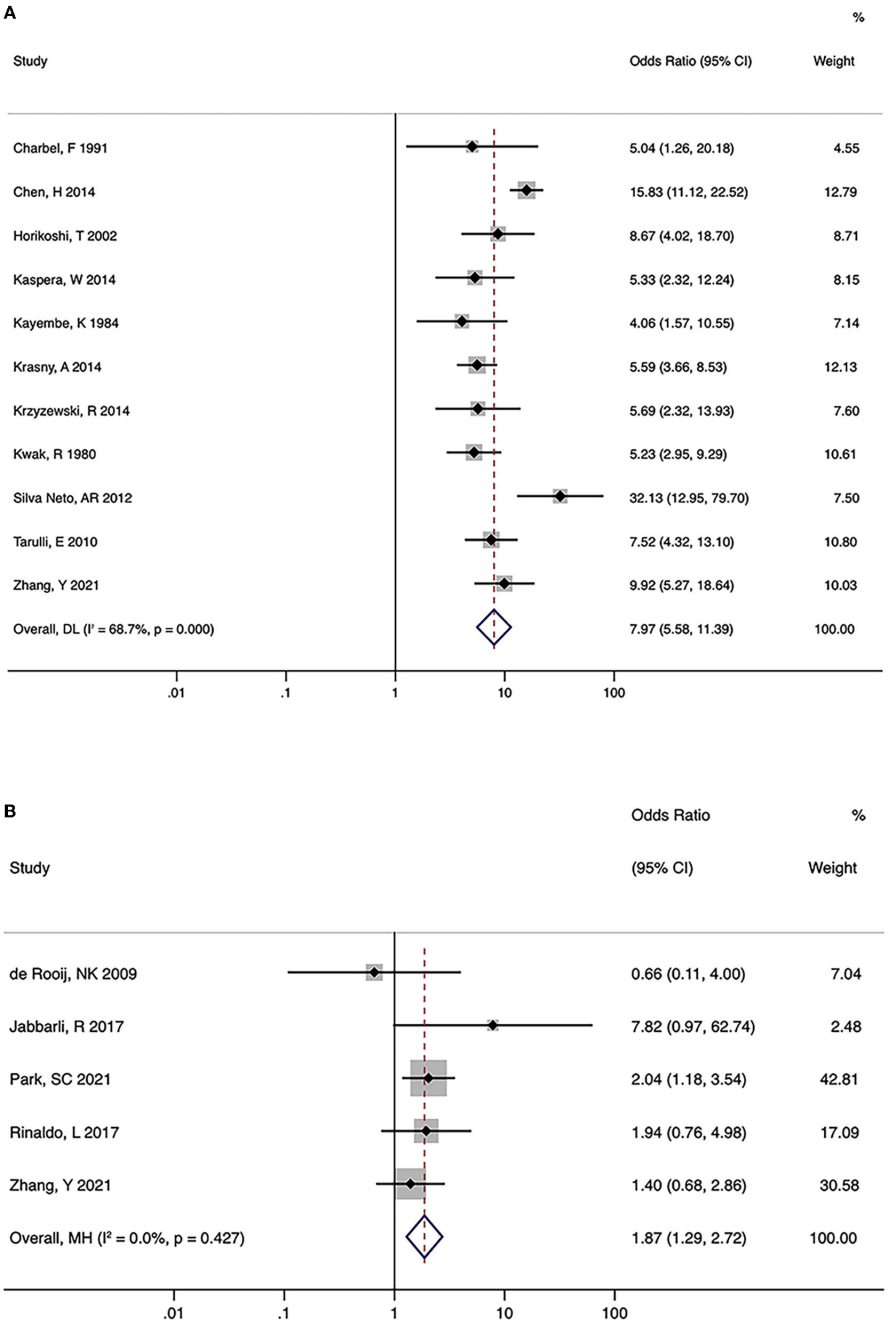
Summary odds ratios of hypoplastic/aplastic A1 and anterior communicating artery (AcomA) aneurysms. **(A)** AcomA aneurysms vs. healthy controls **(B)** ruptured vs. unruptured AcomA aneurysms.

A total of 15 studies investigated the association between hypoplastic/aplastic A1 and the formation and rupture of AcomA aneurysms (Figures 2A, B). Moreover, 11 trials on the formation of AcomA aneurysms showed significant between-study heterogeneity (I^2^ = 68.7%, *P* < 0.001). Pooled analysis using random-effects model showed a prominent relationship between hypoplastic/aplastic A1 and the formation of AcomA aneurysms (OR (95% CI) = 7.97 (5.58, 11.39), *P* < 0.001). All studies showed a significant higher incidence of hypoplastic/aplastic A1 in patients with AcomA aneurysms than in healthy controls. In addition, pooled analysis of five studies showed a higher prevalence of hypoplastic/aplastic A1 in ruptured AcomA aneurysms than in unruptured ones, with no significant between-study heterogeneity (OR (95% CI) = 1.87 (1.29, 2.72), *P* < 0.001; I^2^ = 0.0%, *P* = 0.427).

Significant heterogeneity was seen in studies on the formation of AcomA aneurysms; hence, subgroup analysis was performed to explore potential sources (Table 3). After stratifying the studies by location (Asian or Western) and study design (case-control or cross-sectional study), the significant relationship between hypoplastic/aplastic A1 and the formation of AcomA aneurysms remained. After stratifying by study design, the heterogeneity of each group became non-significant (I^2^ <50%, *P* > 0.05), suggesting that the study design was a source of heterogeneity.

**Table 3:** Subgroup analyses of hypoplastic/aplastic A1 and anterior communicating artery (AcomA) aneurysm formation.

**Outcomes**	**No. of study**	**OR (95%CI)**	***P*-value**	**Heterogeneity test**	

				**I**^2^ **(%)**	*P*_H_
Overall	11	7.97 (5.58, 11.39)	<0.001	68.7	<0.001
Area					
Asian	5	8.33 (4.91, 14.13)	<0.001	73.2	0.005
Western	6	7.59 (4.66, 12.37)	<0.001	60.9	0.025
Design					
Case-control study	10	7.16 (5.25, 9.75)	<0.001	45.4	0.057
Cross-sectional study	1	15.83 (11.12, 22.52)	<0.001	NA	NA

NA, not available.

In the sensitivity analysis of both the formation and rupture of AcomA aneurysms, when a named study was removed, the pooled estimates remained significant (*P* < 0.05), suggesting that none of the studies had a large influence on the overall estimate.

No indication of publication bias for the studies on the formation of AcomA aneurysms was found using the Egger test (*p* = 0.373). However, due to the limited number of studies, the Egger test was not performed for studies on rupture.

### FTP and PcomA aneurysms

**Figure 3:**
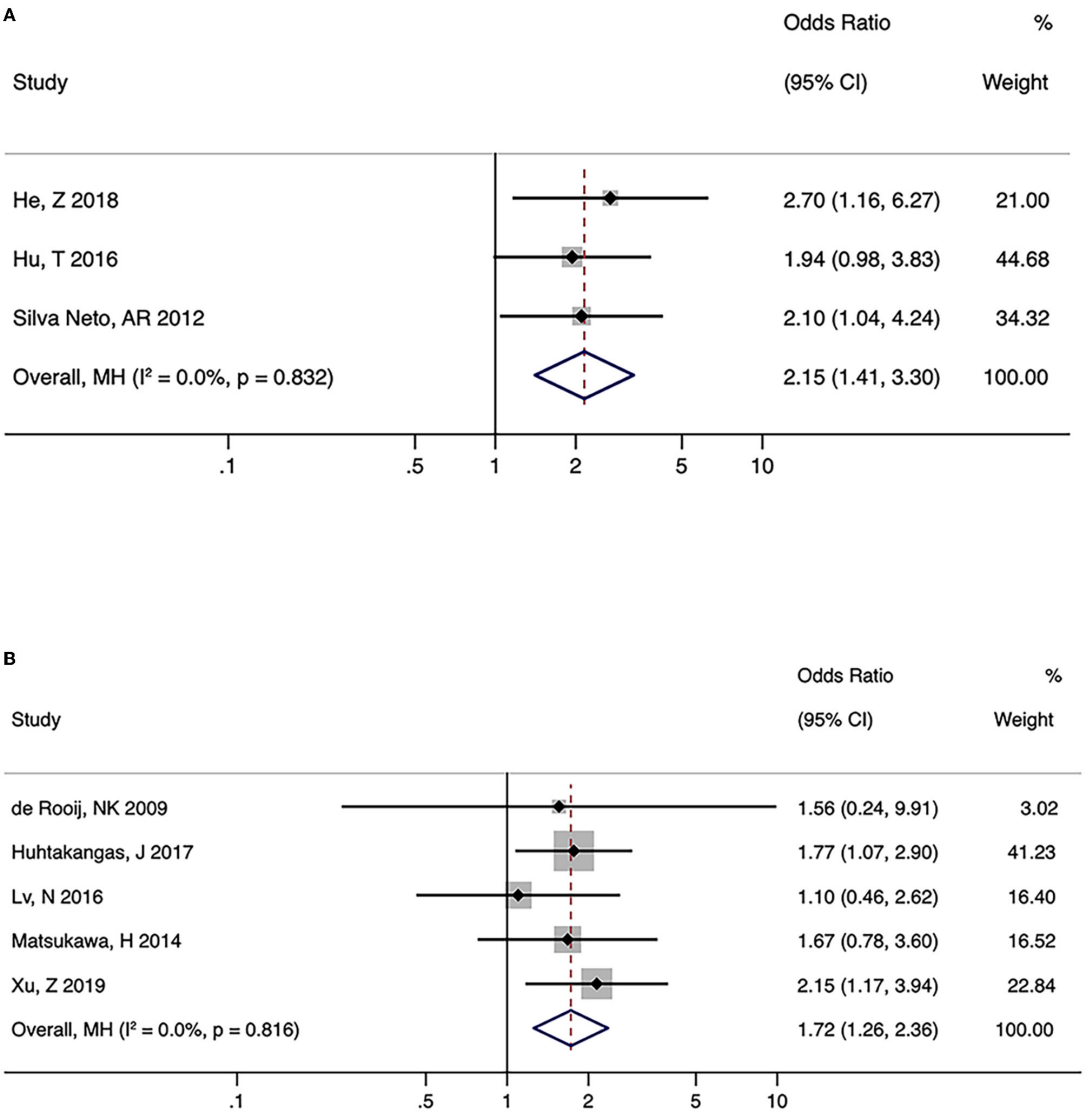
Summary odds ratios of fetal-type posterior cerebral artery (FTP) and posterior communicating artery (PcomA) aneurysms. **(A)** PcomA aneurysms vs. controls **(B)** ruptured vs. unruptured PcomA aneurysms.

There were three and five studies on the relationship between FTP and the formation and rupture of PcomA aneurysms, respectively. No significant heterogeneity was observed (formation: I^2^ = 0.0%, *P* = 0.832; rupture: I^2^ = 0.0%, *P* = 0.816). Pooled analysis using the fixed-effects model indicated a significant association between FTP and both the formation and rupture of PcomA aneurysms (formation: OR (95% CI) = 2.15 (1.41, 3.30), *P* < 0.001; rupture: OR (95% CI) = 1.72 (1.26, 2.36), *P* < 0.001) (Figures 3A, B). The sensitivity analysis of the two groups suggested stable pooled estimates after removing a named study in turn. However, due to the limited number of studies, Egger test was not performed.

### Hypoplastic/aplastic PcomA and PcomA aneurysms

There were two studies on hypoplastic/aplastic PcomA, in which one focused on the formation of PcomA aneurysms and the other on rupture. The OR for hypoplastic/aplastic PcomA and PcomA aneurysm formation was 8.5 (95% CI = 1.9 to 38.1), and that for PcomA rupture was 0.6 (95% CI = 0.1 to 4.1).

## Discussion

This systematic review and meta-analysis investigated the association between COW variations and the formation and rupture of AcomA and PcomA aneurysms. For hypoplastic/aplastic A1 and AcomA aneurysms, a strong association was identified with AcomA aneurysm formation (OR = 7.97), and a moderate association with aneurysm rupture (OR = 1.87). FTP is a risk factor for both the formation and rupture of PcomA aneurysms. However, evidence on hypoplastic/aplastic PcomA and PcomA aneurysms is lacking.

### COW variations and aneurysm formation

COW variations play an important role in the formation of intracranial aneurysms, as the incidence of variations was significantly higher in patients with aneurysms than in controls (34). This association arises from hemodynamic changes, because the elevated wall shear stress caused by COW variations may be a predisposition to aneurysm formation (6).

Numerous imaging and cadaver studies described the coincidence of hypoplastic/aplastic A1 and AcomA aneurysms (10–14, 20, 30, 32–35). In this systematic review and meta-analysis, the prevalence of hypoplastic/aplastic A1 ranged from 24.3 to 69.5% in patients with AcomA aneurysms. The difference in prevalence was due to the limited sample size and different definitions of A1 segment hypoplasia and aplasia (26). With one dominant A1 perfusing the bilateral A2s (30), the increased blood flow across the AcomA elevated the hemodynamic stress dramatically at the artery wall, which may contribute to the formation of AcomA aneurysms (8).

The incidence of FTP among patients with PcomA aneurysms ranged from 17.1 to 45.8%. FTP is formed during embryogenesis. After the internal carotid arteries (ICA) give rise to each arterial segment of COW, the blood of the P2-segment of the posterior cerebral artery (PCA-P2) is equally supplied by the P1-segment of the posterior cerebral artery (PCA-P1) and PcomA. Subsequently, when the occipital lobe develops rapidly, either the PCA-P1 or PcomA enlarges to maintain its blood supply. Considering this, FTP refers to the configuration in which the PcomA outsizes the ipsilateral PCA-P1 (36). When the blood supply to the PCA is predominantly from the ICA-PcomA, blood flow increases in the ICA (37), leading to elevated wall shear stress at the ICA-PcomA junction, which may induce the formation of PcomA aneurysms (9).

### COW variations and aneurysm rupture

The size and location of intracranial aneurysms were significantly correlated with the risk of rupture. Although the International Study of Unruptured Intracranial Aneurysms indicated that small aneurysms (<7 mm) located in the ICA system had a minimal risk of rupture (2), a pooled analysis of six cohort studies observed that AcomA and PcomA aneurysms were associated with a higher risk of rupture, even those that have sizes <7 mm (4). Therefore, reliable predictors for rupture risk are needed for managing patients with AcomA and PcomA aneurysms.

Given its potential effects on wall shear stress, COW configurations might also potentially increase the risk of aneurysm rupture (6). However, the number of studies is limited, and the conclusions were conflicting. One possible reason is that the sample sizes of most studies were limited to one or two hundred, leading to an insufficient number of patients with those COW variations. In this pooled analysis, we confirmed the association between hypoplastic/aplastic A1 and the rupture status of AcomA aneurysms, and FTP and the rupture of PcomA aneurysms.

### Strengths and limitations

First, since the past studies were largely narrative, we chose systematic review and meta-analysis to provide a more comprehensive and unbiased synthesis of relevant studies, and also offered an explicit and exhaustive reporting of the methods used here. By stratifying the variations and site of aneurysms, the localization corresponds to where the hemodynamic stresses were prominently increased, which gives a better understanding of the role of COW configuration on hemodynamics in the development of aneurysms. Second, although there was significant heterogeneity in studies on hypoplastic/aplastic A1 and the formation of AcomA aneurysms, the pooled estimate showed a high correlation strength between the two. In addition, the sensitivity analysis suggested a stable overall estimate. Third, the Egger test performed showed no evidence of publication bias in the meta-analysis, indicating the high credibility of the pooled results.

Nevertheless, this study has several limitations. Only articles published in English were included. Furthermore, the number of studies on aneurysm rupture is limited and are based on small series, which makes it hard to perform subgroup analysis by stratifying the studies by location. Thus, further high-quality research with a large sample size is needed to validate the stability of the results across populations.

### Implications for research and clinical practice

Because of the greater availability of non-invasive imaging techniques, including computed tomography angiography and magnetic resonance angiography, a great number of unruptured intracranial aneurysms are observed. According to our findings, AcomA aneurysms with hypoplastic/aplastic A1 and PcomA aneurysms with FTP require close monitoring and can be an indication for treatment in addition to other morphological features of aneurysms.

Since COW configurations can influence aneurysm morphology and endovascular access to aneurysms, the effect of COW variations on the efficacy or complication rate of aneurysm treatment may warrant further investigation (27, 38, 39).

## Conclusions

In summary, COW variations and both the formation and rupture of AcomA and PcomA aneurysms were significantly associated. These findings could increase awareness for the need to improve screening among patients with relevant COW variations, and also help with intervention determination for patients with aneurysms. Further studies with high quality and large sample sizes are needed.

## Data availability statement

The original contributions presented in the study are included in the article/Supplementary material, further inquiries can be directed to the corresponding author.

## Author contributions

LF and H-JM contributed to data analysis and manuscript drafting. D-DZ, FH, and Y-CZ revised the data for the intellectual contents. FH and Y-CZ were responsible for study conception and interpretation. All authors contributed to the article and approved the submitted version.
