# Supplementary material for: Anatomical variations in the Circle of Willis and the formation and rupture of intracranial aneurysms: A systematic review and meta-analysis
Source: Front Neurol. 2023 Jan 16;13:1098950. doi: 10.3389/fneur.2022.1098950 (PMC9885143; doi:10.3389/fneur.2022.1098950)
Supplement: Supplementary file 1 [file Table_1.docx]

**SUPPLEMENTAL MATERIAL**

**Article Title:** Anatomical variations in the Circle of Willis and the formation and rupture of intracranial aneurysms: a systematic review and meta-analysis

**Table S1.** PubMed search strategy

**Table S2.** Embase search strategy

**Table S3.** Web of Science search strategy

**Table S4.** Quality assessment of the included cross-sectional studies

**Table S5.** Quality assessment of the included cohort studies

**Table S6.** Quality assessment of the included case-control studies

**Table S1. PubMed search strategy**

| Search | Query | Items found |
| --- | --- | --- |
| #1 | (intracranial Aneurysm[MeSH] OR intracranial aneurysm*[tiab]) | 34065 |
| #2 | ("variation"[tiab] OR "variations"[tiab] OR atypical[tiab] OR abnormal*[tiab] OR anomal*[tiab] OR unusual[tiab] OR incomplete[tiab] OR hypoplasia[tiab] OR aplasia[tiab] OR anatom*[tiab] OR asymmetry[tiab]) | 2544776 |
| #3 | (circle of Willis[Mesh]) OR (circle of willis[tiab]) | 3601 |
| #4 | "anterior cerebral artery"[MeSH] OR "anterior cerebral artery"[tiab] OR Anterior communicating artery [tiab] | 6110 |
| #5 | "carotid artery, internal"[MeSH] OR "internal carotid artery"[tiab] | 29674 |
| #6 | "posterior cerebral artery"[MeSH] OR "posterior cerebral artery"[tiab] OR "posterior communicating artery"[tiab] | 5135 |
| #7 | #3 OR #4 OR #5 OR #6 | 39940 |
| #8 | #1 AND #2 AND #7 | 1773 |

**Table S2. Embase search strategy**

| Search | Query | Items found |
| --- | --- | --- |
| #1 | ('intracranial aneurysm'/exp OR 'intracranial aneurysm') | 33834 |
| #2 | ('variation'/exp OR 'variation' OR 'variations' OR atypical OR abnormal* OR anomal* OR unusual OR incomplete OR 'hypoplasia'/exp OR hypoplasia OR 'aplasia'/exp OR aplasia OR anatom* OR 'asymmetry'/exp OR asymmetry) | 3334684 |
| #3 | ('circle of willis'/exp OR 'circle of willis':ti,ab OR 'anterior cerebral artery'/exp OR 'anterior cerebral artery':ti,ab OR 'anterior communicating artery'/exp OR 'anterior communicating artery:ti,ab' OR 'internal carotid artery'/exp OR 'internal carotid artery':ti,ab OR 'posterior cerebral artery'/exp OR 'posterior cerebral artery':ti,ab OR 'posterior communicating artery':ti,ab) | 72805 |
| #4 | #1 AND #2 AND #3 | 2085 |

**Table S3. Web of Science search strategy**

| Search | Query | Items found |
| --- | --- | --- |
| #1 | intracranial Aneurysm (All Fields) | 21341 |
| #2 | "variation" OR "variations" OR atypical OR abnormal* OR anomal* OR unusual OR incomplete OR hypoplasia OR aplasia OR anatom* OR asymmetry (All Fields) | 3712042 |
| #3 | "circle of Willis" OR "anterior cerebral artery" OR "internal carotid artery" OR "posterior cerebral artery" OR "posterior communicating artery" OR "Anterior communicating artery" (All Fields) | 23359 |
| #4 | #1 AND #2 AND #3 | 1074 |

**Table S4. Quality assessment of the included cross-sectional studies**

| Study | A | B | C | D | E | F | G | H | I | J | K | Total Scores |
| --- | --- | --- | --- | --- | --- | --- | --- | --- | --- | --- | --- | --- |
| Chen, H 2014 | 1 | 1 | 1 | 1 | 0 | 0 | 1 | 0 | 0 | 1 | 0 | 6 |
| He, Z 2018 | 1 | 1 | 1 | 1 | 0 | 1 | 1 | 0 | 0 | 1 | 0 | 7 |
| Huhtakangas, J 2017 | 1 | 1 | 1 | 1 | 0 | 0 | 1 | 0 | 0 | 1 | 0 | 6 |
| Lv, N 2016 | 1 | 1 | 1 | 1 | 0 | 0 | 1 | 1 | 0 | 1 | 0 | 7 |
| Matsukawa, H 2014 | 1 | 1 | 1 | 1 | 1 | 1 | 1 | 1 | 0 | 1 | 0 | 9 |
| Xu, Z 2019 | 1 | 1 | 1 | 1 | 0 | 0 | 1 | 0 | 0 | 1 | 0 | 6 |

A: Define the source of information (survey, record review);

B: List inclusion and exclusion criteria for exposed and unexposed subjects (cases and controls) or refer to previous publications;

C: Indicate time period used for identifying patients;

D: Indicate whether or not subjects were consecutive if not population-based;

E: Indicate if evaluators of subjective components of study were masked to other aspects of the status of the participants;

F: Describe any assessments undertaken for quality assurance purposes (e.g., test/retest of primary outcome measurements);

G: Explain any patient exclusions from analysis;

H: Describe how confounding was assessed and/or controlled;

I: If applicable, explain how missing data were handled in the analysis;

J: Summarize patient response rates and completeness of data collection;

K: Clarify what follow-up, if any, was expected and the percentage of patients for which incomplete data or follow-up was obtained.

**Table S5. Quality assessment of the included cohort studies**

| **Study** | **Representati- veness of the exposed cohort** | **Selection of the unexposed cohort** | **Ascertainment of exposure** | **Outcome of interest not present at start of study** | **Control for important factor or additional factor** | **Outcome assessment** | **Follow-up long enough for outcomes to occur** | **Adequacy of follow-up of cohorts** | **Total quality scores** |
| --- | --- | --- | --- | --- | --- | --- | --- | --- | --- |
| Jabbarli, R 2017 | ☆ | ☆ | ☆ | -- | -- | ☆ | ☆ | ☆ | 6 |
| Park, SC 2021 | ☆ | ☆ | ☆ | -- | ☆ | ☆ | ☆ | ☆ | 7 |
| Rinaldo, L 2017 | ☆ | ☆ | ☆ | -- | ☆ | ☆ | ☆ | ☆ | 7 |

**Table S6. Quality assessment of the included** **case-control studies**

| **Study** | **Representativeness of the cases** | **Case definition adequate** | **Ascertainment of exposure** | **Same method of ascertainment for cases and controls** | **Control for important factor or additional factor** | **Selection of Controls** | **Definition of Controls** | **Non- Response rate** | **Total quality scores** |
| --- | --- | --- | --- | --- | --- | --- | --- | --- | --- |
| Charbel, F 1991 | ☆ | ☆ | ☆ | ☆ | -- | -- | ☆ | -- | 5 |
| de Rooij, NK 2009 | ☆ | ☆ | ☆ | ☆ | -- | -- | ☆ | ☆ | 6 |
| Horikoshi, T 2002 | ☆ | ☆ | ☆ | ☆ | -- | -- | ☆ | ☆ | 6 |
| Hu, T 2016 | ☆ | ☆ | ☆ | ☆ | ☆ | -- | ☆ | ☆ | 7 |
| Kaspera, W 2014 | ☆ | ☆ | ☆ | ☆ | -- | -- | ☆ | ☆ | 6 |
| Kayembe, K 1984 | ☆ | ☆ | ☆ | ☆ | -- | -- | ☆ | ☆ | 6 |
| Krasny, A 2014 | ☆ | ☆ | ☆ | ☆ | -- | -- | ☆ | ☆ | 6 |
| Krzyzewski, R 2014 | ☆ | ☆ | ☆ | ☆ | -- | -- | ☆ | ☆ | 6 |
| Kwak, R 1980 | ☆ | ☆ | ☆ | ☆ | -- | -- | ☆ | ☆ | 6 |
| Silva Neto, AR 2012 | ☆ | ☆ | ☆ | ☆ | -- | -- | ☆ | ☆ | 6 |
| Tarulli, E 2010 | ☆ | ☆ | ☆ | ☆ | -- | -- | ☆ | ☆ | 6 |
| Zhang, Y 2021 | ☆ | ☆ | ☆ | ☆ | ☆ | -- | ☆ | ☆ | 7 |
